# Supplementary material for: Geriatric Assessment in a Primary Care Environment: A Standardized Patient Case Activity for Interprofessional Students
Source: MedEdPORTAL. 2019 Oct 18;15:10844. doi: 10.15766/mep_2374-8265.10844 (PMC6944254; doi:10.15766/mep_2374-8265.10844)
Supplement: Supplementary file 1 — A. Logistics.docx B. Case Briefing.docx C. Student Instructions.docx D. IPE Feedback Rubric.docx E. SP Recruiting Criteria.docx F. SP Case Development Tool.docx G. Faculty Instructions and Debriefing Guide.docx H. Potential Discipline-Specific Learning Objectives.docx [file mep-15-10844-s001.zip › _Educational_Summary_Report_10844.pdf]

MedEdPORTAL® | The AAMC Journal of Teaching and Learning Resources

Original Publication

Open Access

Geriatric Assessment in a Primary Care Environment: A Standardized Patient Case Activity for Interprofessional Students

Kelly Karpa, RPh, PhD*, Molly Graveno, Megan Brightbill, RDH, MEd, PHDHP, Gina Fox, OTD, OTR/L, Shawnee Kelly, MS, RDN, LDN, FAND, Erik Lehman, MS, Angela Salvadia, EdD, OTR/L, Tanya Shaw, MEd, Dylan Smith, Matthew Walko, PT, DPT, CCS, Lisa Sherwood, MD

*Corresponding author: kjd136@psu.edu

Abstract

Introduction: Given the aging population and the benefits of comprehensive geriatric assessment to this subset of patients, an interprofessional education training approach may be advantageous for learners from a number of different health professions. **Methods:** Through intercollegiate collaborations involving seven different colleges, an interprofessional simulation using standardized patients was developed and instituted for learners in medicine, nursing, pharmacy, occupational therapy, physical therapy, dental hygiene, and dietitian programs. Herein, we describe the design of the simulation experience and examine its impact on students, as assessed primarily via written reflective comments provided via exit slips at the conclusion of the activity. **Results:** Of the 340 student participants, 83% submitted exit slips describing something gained from the interprofessional session that would not have occurred if students had completed the activity with only students from their own discipline. Three key themes were identified from these reflections: new understanding of roles and responsibilities of other disciplines, new knowledge or skills pertaining to geriatric assessments, and the value of teamwork. **Discussion:** Reflective comments from students regarding the interprofessional experience are evidence of this initiative's benefits, which include increasing knowledge of geriatric medical and allied health-provided care and attainment of interprofessional competencies.

Keywords
Interprofessional Education, Comprehensive Geriatric Assessment, Primary Care, Standardized Patient, Simulation

Educational Objectives

By the end of this activity, learners will be able to:

1. Work collaboratively with others who provide care to deliver preventive and/or health services.
2. Describe at least one aspect of another profession's roles/responsibilities or scope of practice that they did not know prior to the activity.
3. Communicate discipline-specific knowledge to other members of the health care team with confidence and clarity.

Citation:
Karpa K, Graveno M, Brightbill M, et al. Geriatric assessment in a primary care environment: a standardized patient case activity for interprofessional students. *MedEdPORTAL*. 2019;15:10844.
https://doi.org/10.15766/mep_2374-8265.10844

Introduction

The proportion of geriatric patients in the United States has increased substantially over the past decade and is expected to continue growing over the next 30 years. In 2016, individuals older than 65 years constituted 15% of the total population, and this number is expected to reach 25% by the year 2050.[1] In addition to the growing number of geriatric patients, the National Ambulatory Medical Care Survey reported that patients older than 65 years account for approximately 31% of all outpatient office visits.[2] This shift in population demographics presents a clear need to adequately prepare health profession learners to provide care for geriatric patients. Geriatric populations often present unique challenges. For example, accurate diagnoses can be difficult due to atypical presentation of symptoms. Additionally, elderly patients require assessments for conditions that include fall risk, mobility disorders, and urinary incontinence, as well as cognitive impairment.[3] Furthermore, considering that elderly patients tend to have a greater number of chronic illnesses (40% have three or more chronic disease states), thorough assessments and appropriate therapeutic modalities

Copyright © 2019 Karpa et al. This is an open-access publication distributed under the terms of the Creative Commons Attribution license.

may become complex and challenging.[2] To adequately address the needs of geriatric populations, comprehensive geriatric assessments are necessary to account for the many facets that can impact appropriate care delivery to this vulnerable population.[4-7]

At Pennsylvania State University College of Medicine, there is a long history of training medical students in the skills and tools needed to perform geriatric assessments using standardized patients (SPs); however, until recently, this training has taken place in a uniprofessional manner. Of late, the decision was made to provide a similar learning opportunity using an interprofessional education (IPE) paradigm. IPE provides students from two or more professional programs the opportunities to learn with, from, and about each other for the purposes of improving patient care. We herein describe an IPE experience that relies on SPs in a simulated environment and draws from the theoretical framework of Kolb.[8] According to Kolb's theory of learning, students learn best through concrete experiences, reflective observations (watching), abstract conceptualization (thinking), and active experimentation (doing). We selected this active learning strategy to promote a deeper understanding of the needs of and assessments for geriatric patients, encourage higher-level critical thinking skills, and support skill development so that the content is seen as valuable and directly applicable to education and future health care practice. Furthermore, by engaging in the simulated activity in an interprofessional manner, learners can peer teach from their own discipline-specific perspectives while simultaneously engaging in the Interprofessional Education Collaborative (IPEC) competencies of communication, collaboration, and teamwork.[9] Students practice these skills in a low-risk environment, with time for reflection and feedback, before transitioning to authentic clinical encounters with higher-stakes demands on the team.

Several simulation activities exist in the literature that focus on differing aspects relevant to care of geriatric patients. However, preexisting cases tend to focus on very specific aspects of geriatric medicine (e.g., unintentional weight loss, fall risk, and oral health care) or involve limited interprofessional interactions.[10-16] However, our scenario differs by incorporating health professions students from seven different areas of health care and, in doing so, offers a broad, comprehensive overview of geriatric care.

Methods

An interprofessional team of individuals representing various health care specialties created an IPE simulation about comprehensive geriatric assessments. The goal of the activity was for health professions students to learn with, from, and about each other in order to promote interprofessional collaboration, teamwork, and discovery of roles of different health care disciplines. We created the scenario with the knowledge and skill level of learners from the following programs in mind: second professional year of medical school, first professional year of nursing school from the Pennsylvania State University College of Nursing, second professional year of occupational therapy from Elizabethtown College, second professional year of physical therapy at Lebanon Valley College, third professional year of pharmacy training from Appalachian College of Pharmacy (distant learners used videoconferencing technologies due to lack of a local pharmacy program), second year of dental hygiene training from Harrisburg Area Community College, and clinical-year dietetic trainees from an internship program at the Milton S. Hershey Medical Center.

Each discipline contributed to development of the case, as well as discipline-specific learning objectives for students to refine during the activity. For example, specific medications were selected for the case based on muscle pain/fall risk/cognitive issues explicitly for the physical therapy (mobility) and occupational therapy (cognitive) assessments. Additionally, the specific heights and weights for the male and female SPs were deliberately crafted by dietitians for their students' nutritional assessments. In addition, faculty from each professional program jointly developed common interprofessional learning objectives based on the IPEC competencies.[9] The endeavor was a low-stakes, formative learning activity, with individual feedback provided to each student from a faculty facilitator using a rubric that was developed to assess student achievement of IPE competencies. Prior to participation, students read articles about comprehensive geriatric assessments and medication safety and watched videos pertaining to denture care and mobility assessments.[17-20]

Appendix A describes logistical information, including props needed to prepare the simulation room for an outpatient comprehensive geriatric clinic. Each session was 3 hours in length: a 30-minute period for student introductions, establishment of roles, and plans for the patient encounter; 2 hours for student teams to evaluate the SP; and 30 minutes for a faculty-led debrief. After the 2-hour simulation, SPs were permitted to "de-role" by exiting the room briefly (e.g., for a water break) but returned after a few minutes to participate in the debrief when engaged by the faculty member (e.g., "How did the team demonstrate respect for concerns you might have as a

Copyright © 2019 Karpa et al. This is an open-access publication distributed under the terms of the Creative Commons Attribution license.

patient? Was there anything that you experienced that would be concerning/frightening/irritating to an actual patient?").

For the activity, medical students were kept in advising groups per faculty request, whereas other students were randomly added to teams such that each contained learners according to the following distribution: five medicine, two nursing, one to two occupational therapy, one physical therapy, and one pharmacy. Nearly every group also had a dental hygienist, but only eight groups had a dietitian. Each student participated in only one encounter, and each SP portrayed a geriatric patient once per day. Fourteen separate simulation rooms ran simultaneously during the first day (14 SPs), and 16 rooms ran concurrently during the second day (16 SPs). Additional personnel included at least one faculty member facilitator per room who provided limited guidance to students during the encounter but was critical for debriefing the learners and offering feedback to each student following the session. Every room had a physician facilitator (who was also a student advisor); many rooms also had cofacilitators representing faculty from across the other disciplines.

Facilitator training was 2 hours in length. During this time, background information about IPE, learning objectives for the session, and logistical information were discussed. Additionally, in all of our IPE facilitator training sessions, two videos were shown that had been recorded previously, depicting student volunteer actors portraying exemplary versus unprofessional IPE behaviors. The videos were used to train facilitators to apply the feedback rubric to the collaborative behaviors exhibited by students during the IPE activity. Also discussed during training were examples of different ways to ask debriefing questions (e.g., open-ended, closed, boomerang, direct, advocacy inquiry) and the Gather-Analyze-Summarize debriefing framework. These methods were customarily used by our simulation center.

Prior to the activity, students were invited to complete an online interprofessional self-assessment questionnaire (not included; see Lockeman et al.[21] for details; odd-numbered questions assessed the interprofessional Interactions domain, and even-numbered questions assessed the Values domain). Patient background information was provided to set the stage for the activity, as outlined in Appendix B (information in parts 1 and 2 of Appendix B is gender specific; students received only the part that correlated to the SP's gender in their specific room). Students received additional instructions, as found in Appendix C, that outlined general directions, time limits, and expectations for the session.

During the activity, facilitators used a rubric (Appendix D) to evaluate interprofessional collaborative behaviors and provided individual feedback to learners. Using a rubric developed previously by Lie et al.[22] as a guide, all faculty involved with developing this activity contributed to the creation of this tool, which was used to provide feedback and constructive comments to students relative to their demonstration of IPE competency areas that correlated with session-specific objectives. Numeric ratings of 1 indicated unsatisfactory behaviors, ratings of 2 reflected that students were working toward competency, and ratings of 3 indicated mastery of that competency.

For the activity, staff at our clinical simulation center identified SPs based on their age range approximating that of a geriatric patient. A total of 16 SPs were trained. Appendix E depicts the recruitment criteria and training methodology for portrayal of the geriatric patient. SPs maintained their respective roles for 2 hours. Appendix F outlines in detail how the SPs were to act and respond during the scenario. SPs were trained by faculty who had completed a weeklong instructor simulation course that included training SPs for OSCE experiences.

During the activity, facilitators kept individual sessions running on time. Each experience occurred over 180 minutes, which consisted of a 30-minute preparation period for student introductions and discussions to determine designated roles during the encounter, 2 hours for interaction with the SP, and a 30-minute debrief led by a facilitator. Facilitation materials can be found in Appendix G to guide faculty facilitators regarding the session time line and debriefing around interprofessional competencies following the activity. Discipline-specific learning objectives, to be reinforced within individual disciplines, are included for reference in Appendix H.

After the interprofessional encounter, students were invited to complete the voluntary online interprofessional self-assessment questionnaire again, rate the extent to which the activity met learning objectives, and provide general feedback about their experiences. Due to inherent difficulties capturing student input via voluntary electronic evaluative questionnaires, on the day of the interprofessional activity learners were asked to complete exit slips at the conclusion of the event. Exit slips were written student responses that served as a quick, informal assessment gauging students' understanding of the material or the impact that the activity had. Specifically, students were asked to describe one thing learned as a result of the IPE event that would not have been learned if the activity had been conducted uniprofessionally, with only their discipline present.

Copyright © 2019 Karpa et al. This is an open-access publication distributed under the terms of the Creative Commons Attribution license.

Statistical analysis of paired and unpaired data collected from the online questionnaires was performed using the Wilcoxon signed rank test to compare medians of the interprofessional competency self-assessment tool before versus after activity, with significance set at $p < .05$. Means and standard deviations evaluated students' ratings regarding the extent to which the activity met intended learning objectives and facilitators' ratings of students' performance using the feedback rubric. In addition, three researchers used a directed content analysis approach to electronically code each exit slip comment. Initial coding was based on IPEC competencies but expanded when new concepts, not directly tied to interprofessional competencies, emerged. Collectively, every student response was independently coded by each researcher; discrepancies between codes were discussed among the three researchers until consensus was reached.

This activity was exempt from investigational review board oversight as determined by the Human Subjects Protection Office at the Penn State Health Milton S. Hershey Medical Center.

Results

A total of 340 students participated in this interprofessional activity, including learners from the following programs: medicine ($n = 142$), nursing ($n = 55$), occupational therapy ($n = 48$), physical therapy ($n = 36$), pharmacy ($n = 30$), dental hygiene ($n = 21$), and dietitian ($n = 8$). Nineteen percent ($n = 63$) of students opted to complete the voluntary IPEC self-assessment prior to the activity, but only 15 students completed it after the activity. This resulted in 10 respondents (six pharmacy, two physical therapy, two nursing) who were able to be matched with preactivity responses using unique identifiers. Median results for both the paired and unpaired responses are shown in Table 1. For paired responses, no individual items had a statistically significant change when comparing the two time points; however, cumulatively, there was a statistically significant

Table 1. Pre- and Postactivity Responses to the Interprofessional Self-Assessment Questionnaire

	Paired			Unpaired		
Question	Preactivity Median (n = 10)	Postactivity Median (n = 10)	p	Preactivity Median (n = 68)	Postactivity Median (n = 18)	p
I am able to . . .						
1. Choose communication tools and techniques that facilitate effective team interactions.	4.0	4.0	.38	4.0	4.0	.10
2. Place interests of patients at the center of interprofessional health care delivery.	4.0	5.0	.13	5.0	5.0	.11
3. Engage other health professionals in shared problem solving appropriate to the specific care situation.	4.0	5.0	.13	4.0	5.0	.004
4. Respect the privacy of patients while maintaining confidentiality in the delivery of team-based care.	5.0	5.0	1.00	5.0	5.0	.35
5. Inform care decisions by integrating the knowledge and experience of other professions appropriate to the clinical situation.	4.0	4.5	.31	4.0	4.0	.023
6. Embrace the diversity that characterizes the health care team.	4.5	5.0	.50	4.0	5.0	.03
7. Apply leadership practices that support effective collaborative practice.	4.0	5.0	.31	4.0	5.0	.002
8. Respect the cultures and values of other health professions.	5.0	4.5	1.00	5.0	5.0	.73
9. Engage other health professionals to constructively manage disagreements about patient care.	4.0	5.0	.02	4.0	5.0	.001
10. Develop a trusting relationship with other team members.	4.0	5.0	.13	4.0	5.0	.06
11. Use strategies that improve the effectiveness of interprofessional teamwork and team-based care.	4.0	5.0	.25	4.0	5.0	.007
12. Demonstrate high standards of ethical conduct in my contributions to team-based care.	4.5	4.5	1.00	4.0	5.0	.20
13. Use available evidence to inform effective teamwork and team-based practices.	4.0	5.0	.13	4.0	5.0	.003
14. Act with honesty and integrity in relationships with other team members.	4.5	5.0	.50	4.0	5.0	.16
15. Understand responsibilities and expertise of other health professions.	4.0	5.0	.25	4.0	5.0	.049
16. Maintain competence in my own profession appropriate to my level of training.	4.5	5.0	.50	4.0	5.0	.047
Cumulative Interprofessional Interactions domain (odd-numbered questions)	4.1	4.8	.04	4.00	4.75	.001
Cumulative Interprofessional Values domain (even-numbered questions)	4.6	4.9	.13	4.38	4.88	.060

Copyright © 2019 Karpa et al. This is an open-access publication distributed under the terms of the Creative Commons Attribution license.

increase observed in Interactions domain responses (e.g., odd-numbered questions) when compared before and after the activity.

At the conclusion of the activity, 18 students logged into an online evaluation of the session and rated the extent to which they were in agreement that the activity met intended objectives. Mean responses are depicted in Table 2. On feedback rubrics that facilitators used to provide comments to individual learners, few students received unsatisfactory ratings in any area. For those who did, the collaboration competency was most often implicated; in this regard, facilitator comments suggested that students needed to either interact more with the group or allow the group more shared decision making and not take over for the group. Most students were rated as working toward competency or having attained competency in the areas related to communication, collaboration, and role delineation that were assessed (Table 3).

Based on exit slip feedback from 283 students, participation in the geriatric assessment activity was a rewarding experience, with three central themes emerging. As a result of the simulation, students had a better understanding of roles and responsibilities of other disciplines, they gained new knowledge and skills pertaining to comprehensively assessing geriatric patients, and they valued the teamwork that was necessary across disciplines to optimize patient care and outcomes.

Specifically, student participants reflected on learning the roles of different disciplines with regard to taking specific aspects of patient history, answering patient questions, assessments/examinations performed, and roles in safe home care for patients. The amount of role overlap existing between professions was new to many students. Some of these newfound realizations regarding roles of health care providers were empowering for the learners, such as the following:

- “[I learned] the importance of each team member’s role. This allowed for me to appreciate the impact other professions have on a patient, and what I can expect from them, and how I can help them”—physical therapy student.
- “[I learned] the overlap of professions, specifically the knowledge gap the pharmacist was able to fill. OT [occupational therapy]/Nutrition provided a great resource in tackling issues related to ADL [activities of daily living]/energy which is a primary concern as a nurse”—nursing student.
- “I do not think I would have realized how big of a role other disciplines play in the care of a patient that is seemingly ‘ours’ as MDs”—medical student.
- “I really enjoyed learning how each occupation plays a role in patient care and it is a relief to know that I do not have to be an expert on everything”—medical student.

Table 2. Rating Scores Pertaining to Learning Objectives (*n* = 18)

Learning Objective Statement	*M*[a]	*SD*
My team worked collaboratively to deliver care and/or preventative services.	4.6	0.61
I learned something new about another professions’ role or scope of practice.	4.8	0.43
I am able to communicate my discipline-specific knowledge to other members of the health care team with confidence and clarity.	4.7	0.49
I can engage other health care professions in shared patient-centered problem solving.	4.6	0.61

[a]Rated on a 5-point Likert scale (1 = *strongly disagree*, 5 = *strongly agree*).

In addition to gaining a better understanding of the roles and responsibilities of other health care providers, students also learned new knowledge from the experience. Many reflected on learning about medications, new skills/cognitive assessments, and oral hygiene:

- “I learned a lot about medications today and what each is necessary for. The patient information lists all medications; however, it does not explain them. Therefore, discussing them with the med and nursing students as well as the pharmacist was really helpful”—occupational therapy student.
- “[I learned about] dental hygiene and that dentures impact diet, how dentures move at night, [and that patients] have to still see dentist”—medical student.
- “[I learned] about the MoCA [Montreal Cognitive Assessment] exam. Dental doesn’t typically deal with any cognitive testings. I had never heard of this”—dental hygiene student.

Students also discovered that teamwork across many different disciplines was essential to optimize patient outcomes. Through this activity, they experienced collaborative care with other disciplines and were able to see the patient through another’s perspective to create a holistic, patient-centered care plan. Examples included the following:

- “I learned how sometimes there is a point where we will not know the answers and other professions can help”—nursing student.
- “I enjoyed watching the other students approach the same patient I was seeing with a different lens and working together with everyone to address many different concerns for the person’s health”—occupational therapy student.

Copyright © 2019 Karpa et al. This is an open-access publication distributed under the terms of the Creative Commons Attribution license.

Table 3. Feedback Rubric Summary Data ($M \pm SD$)

Profession	Communicate With Confidence	Communicate With Clarity	Communicate Effectively	Role Identification	Collaborate Effectively
All professions (n = 326)	2.77 ± 0.42	2.80 ± 0.44	2.74 ± 0.47	2.83 ± 0.41	2.67 ± 0.49
Dental hygiene (n = 19)	2.68 ± 0.48	2.58 ± 0.61	2.74 ± 0.45	2.63 ± 0.60	2.58 ± 0.51
Dietitian (n = 8)	3.00 ± 0.00	3.00 ± 0.00	2.88 ± 0.35	2.60 ± 0.52	2.75 ± 0.46
Medicine (n = 138)	2.78 ± 0.41	2.80 ± 0.40	2.74 ± 0.49	2.88 ± 0.33	2.70 ± 0.46
Nursing (n = 54)	2.59 ± 0.53	2.65 ± 0.62	2.70 ± 0.50	2.78 ± 0.46	2.54 ± 0.54
Occupational therapy (n = 47)	2.87 ± 0.34	2.87 ± 0.40	2.77 ± 0.48	2.85 ± 0.42	2.68 ± 0.52
Pharmacy (n = 24)	2.75 ± 0.44	2.79 ± 0.41	2.54 ± 0.51	2.71 ± 0.55	2.58 ± 0.50
Physical therapy (n = 36)	2.89 ± 0.32	2.97 ± 0.17	2.83 ± 0.38	2.92 ± 0.28	2.81 ± 0.47

Discussion

For most learners, engaging in this activity was a stepping stone in their interprofessional development, placed strategically in the curriculum to bridge classroom-based IPE activities and future simulation activities for which facilitators would no longer be physically present to guide team-based interactions.[23] We believe that after engaging in this geriatric assessment, student participants will be better aware of the challenges facing geriatric patients and the resources that exist in terms of other professions that can help with management.

Considering that IPE was not a new concept to the students, it was not a surprise that few opted to complete the voluntary interprofessional self-assessment tool, nor was it a surprise that with such a low response rate, there was not a more perceptible shift in opinions among the small number of students who completed it both before and after the activity. Nonetheless, when all Interactions domain items were cumulatively evaluated in the paired analysis, there was a statistically significant shift between pre- and postactivity responses, indicating that the activity improved student interactions with other disciplines. Specific objectives within this domain included the ability to "engage other health professionals to constructively manage disagreements about patient care" and "engage other health professionals in shared problem solving appropriate to the specific care situation."

Of students who opted to provide feedback regarding whether the session objectives were attained, the respondents strongly indicated in the affirmative. Considering that unhappy students generally take the time to relay displeasure when new curricular items are initiated, we interpreted the lack of strong negative responses in an encouraging manner. Not only were session objectives reached but exit slip responses indicated that key factors learned by students included roles and responsibilities of other health care providers, how to work as part of a team, and new knowledge/resources pertinent to caring for geriatric patients that they would not have known otherwise.

Given our past experiences as faculty using videoconferencing to bridge distances when building IPE activities, we attempted to use this same technology to involve learners who were physically located 500 miles from our campus. Specifically, 30 pharmacy students connected to each of 30 different classrooms/SPs using videoconferencing technologies because we did not have access to in-person pharmacy learners due to our geographic location. Feedback received from both students and faculty about the videoconferencing aspect of the event revealed that approximately half the time, it worked well, but the other half of the time, the experience could be improved. Connection difficulties, audio challenges, and being/staying engaged at a distance were some of the challenges encountered—some of which might be overcome with the assistance of dedicated information technology support in each classroom, as well as additional faculty development focused on engaging distant learners.

An important lesson regarding creation of student interprofessional teams was recognized. For a variety of reasons, the medical student participants were not randomized for this IPE session. Instead, cohorts of five medical students, along with their faculty advisor (e.g., session facilitator), were joined by the other student learners and, in most groups, a cofacilitator from another discipline. Although this worked well in most instances, two students from nonmedical disciplines reported negative experiences that likely stemmed from difficulties assimilating into an intact group with an already-established history. Although, in actual practice, individuals from different disciplines will always be interacting with other team members who have a prior history of working together, it is probably best not to place that extra burden on learners at this point in their training. In the future, we will randomize the medical students and separate them from their faculty advisors so that there is less tendency for nonmedical learners to feel like outsiders.

It was also discovered during this activity that it is advantageous to provide learners with not only the case in advance but also

Copyright © 2019 Karpa et al. This is an open-access publication distributed under the terms of the Creative Commons Attribution license.

opportunities to interact prior to the IPE event. Historically, prior to other IPE events, we utilized a course management system to upload chart notes/background information in advance and created discussion boards to allow student teams to interact with each other prior to the actual event. For logistical reasons, we were unable to do that prior to this particular activity. It would be advantageous to utilize the online discussion board in the future so that students can meet their team members in advance, begin outlining a plan for the geriatric assessment, and perhaps even role-play or seek a consultation for teams lacking students from less represented disciplines (e.g., dietitians). As noted by Shortridge et al.,[10] providing students with background on the case in advance can help them be more prepared and can alleviate stressors involved with coordinating small groups on a time schedule or technology malfunctions. Furthermore, having interactions and discussions prior to the activity would be a time-saver on the day of the activity, as introductions could be shorter and engaging in the patient assessment could be done in a more directed fashion.

Overall, faculty facilitators offered several positive comments about the activity. Some shared that they had learned things from other disciplines that they were looking forward to incorporating into their own practices. Others admitted to initial skepticism about how the scenario would run and about contributions that some disciplines would be able to make to the case; however, early skeptics admitted to being pleasantly surprised at the level of knowledge and skills that the various disciplines brought to the scenario and how smoothly the event ran. Several had suggestions for other disciplines that potentially could be included in the future, such as psychology, social work, and nurse practitioner and physician assistant programs.

A limitation to interprofessional endeavors can be over- or underrepresentation of specific disciplines. Despite each of the professions engaged in this activity requiring students' participation, there are not equal numbers of students enrolled in each of the programs. IPE is often limited by real-world realities rather than ideal situations. At the same time, the unequal distribution of students is often reminiscent of the professional world. For example, a geriatric patient may have five physicians but only a single pharmacist or physical therapist; as a result, learners must gain confidence in their knowledge and skills and be empowered to share their views and opinions with other team members for successful patient outcomes regardless of whether their profession is equally represented on the care team.

The generalizability of lessons learned and of the impact that this activity had on developing students' interprofessional competencies is limited by the low response rates obtained from pre- and postactivity questionnaires; low response rates are problematic when students opt out of participating in voluntary surveys. Nonetheless, the questionnaire primarily assesses perceptions rather than actual skills. In contrast, student responses on the exit slips support the notion that interprofessional learning occurred, with many citing specific new skill sets and knowledge that they learned as a direct result of the activity being conducted in an interprofessional manner. Based on the reflective responses, this activity clearly addresses IPEC competencies pertaining to roles and teamwork, but other activities are needed to highlight the IPEC competencies of shared values/ethics and interprofessional communication.

Appendices

A. Logistics.docx
B. Case Briefing.docx
C. Student Instructions.docx
D. IPE Feedback Rubric.docx
E. SP Recruiting Criteria.docx
F. SP Case Development Tool.docx
G. Faculty Instructions and Debriefing Guide.docx
H. Potential Discipline-Specific Learning Objectives.docx

All appendices are peer reviewed as integral parts of the Original Publication.

Kelly Karpa, RPh, PhD: Professor, Department of Pharmacology, Pennsylvania State University College of Medicine; Assistant Dean for Interprofessional Education, Pennsylvania State University College of Medicine

Molly Graveno: PharmD Candidate, Duquesne University School of Pharmacy

Megan Brightbill, RDH, MEd, PHDHP: Campus Associate Dean, Academic Affairs, Harrisburg Area Community College

Gina Fox, OTD, OTR/L: Lecturer, Occupational Therapy, Elizabethtown College

Shawnee Kelly, MS, RDN, LDN, FAND: Assistant Teaching Professor, Department of Nutritional Sciences, Pennsylvania State University College of Health and Human Development; Coordinator of Master of Professional Studies in Nutritional Sciences, Department of Nutritional Sciences, Pennsylvania State University College of Health and Human Development

Erik Lehman, MS: Biostatistician, Department of Public Health Sciences, Pennsylvania State University College of Medicine

Copyright © 2019 Karpa et al. This is an open-access publication distributed under the terms of the Creative Commons Attribution license.

Angela Salvadia, EdD, OTR/L: Lecturer, Occupational Therapy, Elizabethtown College

Tanya Shaw, MEd: Education Program Assistant, Pennsylvania State University College of Medicine

Dylan Smith: PharmD Candidate, Nesbitt School of Pharmacy, Wilkes University

Matthew Walko, PT, DPT, CCS: Clinical Assistant Professor, Physical Therapy, Lebanon Valley College

Lisa Sherwood, MD: Assistant Professor, Department of Medicine, Pennsylvania State University College of Medicine

Disclosures
None to report.

Funding/Support
None to report.

Ethical Approval
The Human Subjects Protection Office at Penn State Health Milton S. Hershey Medical Center approved this study.

References

1. Mather M, Jacobsen LA, Pollard KM. Aging in the United States. *Popul Bull.* 2015;70(2):2-18.
2. Rui P, Okeyode T. *National Ambulatory Medical Care Survey: 2015 State and National Summary Tables*. Atlanta, GA: Centers for Disease Control and Prevention; 2015.
3. Seematter-Bagnoud L, Büla C. Brief assessments and screening for geriatric conditions in older primary care patients: a pragmatic approach. *Public Health Rev*. 2018;39:8. https://doi.org/10.1186/s40985-018-0086-7
4. Tinetti ME, Inouye SK, Gill TM, Doucette JT. Shared risk factors for falls, incontinence, and functional dependence: unifying the approach to geriatric syndromes. *JAMA*. 1995;273(17):1348-1353. https://doi.org/10.1001/jama.1995.03520410042024
5. Inouye SK, Studenski S, Tinetti ME, Kuchel GA. Geriatric syndromes: clinical, research, and policy implications of a core geriatric concept. *J Am Geriatr Soc*. 2007;55(5):780-791. https://doi.org/10.1111/j.1532-5415.2007.01156.x
6. Pilotto A, Cella A, Pilotto A, et al. Three decades of comprehensive geriatric assessment: evidence coming from different healthcare settings and specific clinical conditions. *J Am Med Dir Assoc*. 2017;18(2):192.e1-192.e11. https://doi.org/10.1016/j.jamda.2016.11.004
7. Carlson C, Merel SE, Yukawa M. Geriatric syndromes and geriatric assessment for the generalist. *Med Clin North Am*. 2015;99(2):263-279. https://doi.org/10.1016/j.mcna.2014.11.003
8. Kolb DA. *Experiential Learning: Experience as the Source of Learning and Development*. 2nd ed. Upper Saddle River, NJ: Pearson Education; 2015.
9. Interprofessional Education Collaborative Expert Panel. *Core Competencies for Interprofessional Collaborative Practice: Report of an Expert Panel*. Washington, DC: Interprofessional Education Collaborative; 2011.
10. Shortridge A, Steinheider B, Ciro C, Randall K, Costner-Lark A, Loving G. Simulating interprofessional geriatric patient care using telehealth: a team-based learning activity. *MedEdPORTAL*. 2016;12:10415. https://doi.org/10.15766/mep_2374-8265.10415
11. Mulligan R, Gilmer-Scott M, Kouchel D, et al. Unintentional weight loss in older adults: a geriatric interprofessional simulation case series for health care providers. *MedEdPORTAL*. 2017;13:10631. https://doi.org/10.15766/mep_2374-8265.10631
12. Larson C, O'Brien B, Rennke S. GeriWard Falls: an interprofessional team-based curriculum on falls in the hospitalized older adult. *MedEdPORTAL*. 2016;12:10410. https://doi.org/10.15766/mep_2374-8265.10410
13. Kaufman LB, Soto A, Gascon L, et al. Oral health for older adults: an interprofessional workshop for medical students. *MedEdPORTAL*. 2017;13:10572. https://doi.org/10.15766/mep_2374-8265.10572
14. Denham AC. Clinical decision-making for older patients with multiple chronic conditions: a case-based exercise for medical students. *MedEdPORTAL*. 2016;12:10477. https://doi.org/10.15766/mep_2374-8265.10477
15. Dodd M, VanLeit B, Popp J, Herman C, Harris J, Cohen D. Interprofessional geriatric assessment elective for health professional students: a standardized patient case study and patient script. *MedEdPORTAL*. 2014;10:9917. https://doi.org/10.15766/mep_2374-8265.9917
16. Wilson S, Vorvick L. Dyspnea in a hospitalized patient: using simulation to introduce interprofessional collaborative practice concepts. *MedEdPORTAL*. 2016;12:10488. https://doi.org/10.15766/mep_2374-8265.10488
17. Elsawy B, Higgins KE. The geriatric assessment. *Am Fam Physician*. 2011;83(1):48-56.
18. Pretorius RW, Gataric G, Swedlund SK, Miller JR. Reducing the risk of adverse drug events in older adults. *Am Fam Physician*. 2013;87(5):331-336.
19. Timed Up and Go Test (TUG). Physiopedia website. https://www.physio-pedia.com/Timed_Up_and_Go_Test_(TUG). Accessed April 19, 2019.

Copyright © 2019 Karpa et al. This is an open-access publication distributed under the terms of the Creative Commons Attribution license.

20. Dentures. American Dental Association's Mouth Healthy website. https://www.mouthhealthy.org/en/az-topics/d/dentures. Accessed April 19, 2019.

21. Lockeman KS, Dow AW, DiazGranados D, et al. Refinement of the IPEC Competency Self-Assessment survey: results from a multi-institutional study. *J Interprof Care*. 2016;30(6):726-731. https://doi.org/10.1080/13561820.2016.1220928

22. Lie D, May W, Richter-Lagha R, Forest C, Banzali Y, Lohenry K. Adapting the McMaster-Ottawa scale and developing behavioral anchors for assessing performance in an interprofessional team observed structured clinical encounter. *Med Educ Online*. 2015;20:26691. https://doi.org/10.3402/meo.v20.26691

23. Karpa K, Pinto C, Possanza A, et al. Stroke simulation activity: a standardized patient case for interprofessional student learning. *MedEdPORTAL*. 2018;14:10698. https://doi.org/10.15766/mep_2374-8265.10698

Received: February 9, 2019
Accepted: June 21, 2019
Published: October 18, 2019

Copyright © 2019 Karpa et al. This is an open-access publication distributed under the terms of the Creative Commons Attribution license.
