# Supplementary material for: Geriatric Assessment in a Primary Care Environment: A Standardized Patient Case Activity for Interprofessional Students
Source: MedEdPORTAL. 2019 Oct 18;15:10844. doi: 10.15766/mep_2374-8265.10844 (PMC6944254; doi:10.15766/mep_2374-8265.10844)
Supplement: Supplementary file 1 — A. Logistics.docx B. Case Briefing.docx C. Student Instructions.docx D. IPE Feedback Rubric.docx E. SP Recruiting Criteria.docx F. SP Case Development Tool.docx G. Faculty Instructions and Debriefing Guide.docx H. Potential Discipline-Specific Learning Objectives.docx [file mep-15-10844-s001.zip › A. Logistics.docx]

**Appendix A: Logistics**

1. Location of Activity
   1. Pennsylvania State University College of Medicine small-group rooms
2. Setting for the Encounter
   1. Primary care office
3. Materials Required
   1. Props in room
      1. Portable BP cuffs
      2. Thermometer and thermometer covers
      3. Medication list for each SP
      4. SPs need 3x5 cards with dental images depicting mouth sores reminiscent of poorly-fitted dentures to give to students if they wish to look in SP’s mouth^2^
      5. Mini-cog test with instructions – only recall 2 of 3 items, but draw the clock appropriately^3^
      6. MOCA in room for students – SPs respond INCORRECTLY to the executive/visuo-spatial section and the memory/delayed recall section^4^
      7. Depression Screen – Geriatric depression screen and scoring with the SP’s answers circled^5^
      8. Link to FRAX and printed handout of FRAX questions.^6^
      9. Timed Up and Go pdf handout^7^
      10. Pocket guide to Beers List drugs^8^

- 1. Facilitator Packets
     1. Student List: include names, disciplines, facilitator name, co-facilitator name, room number, video conferencing URL link and phone number
     2. Sign-in sheets for facilitators and students specific for each room
     3. Student instructions for the activity
     4. Feedback rubric for facilitators to complete for each student
     5. Appropriate case for male or female SP (printed in either pink or blue) (also provided to students in advance)
     6. An exit slip for each student regarding something learned that wouldn’t have been apparent if the case had been done uni-professionally
     7. Facilitator guide

1. Number of encounters completed by each learner
   1. For this case, each student learner completed a one-time, standardized patient (SP) encounter as part of an interprofessional team, followed by a 30-minute team debrief with a facilitator.
2. Length of each encounter
   1. Student teams were given 30 minutes for initial introductions and discussions to develop a team plan for patient encounter.
   2. Students performed assessments with the patient for a total of 2 hours.
   3. After the 2-hour interaction with the SP, a facilitator led a 30-minute debrief with the students.
   4. After completing the team-based activity, students were invited to complete an online, post-activity self-assessment (the Interprofessional Education Competency Self-Assessment)^1^ and answer reflection questions about the experience.
3. Total number of encounters expected in a session for the SP
   1. SPs had one team encounter per day
4. Start and finish time
   1. 3 hours per team
5. Staffing Requirement: 14 to 16 SP rooms run simultaneously
   1. 1 standardized patient needed per room per day
   2. A minimum of one faculty facilitator per room per day
6. Pre-briefing material given to learners prior to attending session
   1. A copy of the case may be provided to learners in advance (see Appendix B: Case Briefing)
7. Briefing orientation materials used
   1. Student instructions for the session are provided in Appendix C: Student Instructions
8. Describe feedback and or debriefing methods used
   1. A facilitator debriefing guide is found in Appendix H: Faculty Instructions And Debriefing Guide
9. Feedback provided post-activity to learners
   1. An IPE Feedback Rubric was developed and is available in Appendix D: IPE Feedback Rubric

Reference:

1. [Lockeman KS](https://www.ncbi.nlm.nih.gov/pubmed/?term=Lockeman%20KS%5BAuthor%5D&cauthor=true&cauthor_uid=27797633), [Dow AW](https://www.ncbi.nlm.nih.gov/pubmed/?term=Dow%20AW%5BAuthor%5D&cauthor=true&cauthor_uid=27797633), [DiazGranados D](https://www.ncbi.nlm.nih.gov/pubmed/?term=DiazGranados%20D%5BAuthor%5D&cauthor=true&cauthor_uid=27797633), [McNeilly DP](https://www.ncbi.nlm.nih.gov/pubmed/?term=McNeilly%20DP%5BAuthor%5D&cauthor=true&cauthor_uid=27797633), [Nickol D](https://www.ncbi.nlm.nih.gov/pubmed/?term=Nickol%20D%5BAuthor%5D&cauthor=true&cauthor_uid=27797633), [Koehn ML](https://www.ncbi.nlm.nih.gov/pubmed/?term=Koehn%20ML%5BAuthor%5D&cauthor=true&cauthor_uid=27797633), [Knab MS](https://www.ncbi.nlm.nih.gov/pubmed/?term=Knab%20MS%5BAuthor%5D&cauthor=true&cauthor_uid=27797633). Refinement of the IPEC Competency Self-Assessment survey: Results from a multi-institutional study. [*J Interprof Care*.](https://www.ncbi.nlm.nih.gov/pubmed/?term=dow+interprofessional+education+competency+self+assessment) 2016;30(6):726-731.
2. da Silva HF, Martins-Filho PR, Piva MR. Denture-related oral mucosal lesions among farmers in a semi-arid Northeastern Region of Brazil. *Med Oral Patol Oral Cir Bucal.* 2011;16:e742. doi:10.4317/medoral.17081
3. Borson S, Scanlan JM, Chen PJ et al. The Mini‐Cog as a screen for dementia: Validation in a population‐based sample. *J Am Geriatr Soc*. 2003;51:1451–1454. Doi:10.1046/j.1532-5415.2003.51465.x
4. Nasreddine ZS, Phillips NA, Bedirian V, et al. The Montreal Cognitive Assessment, MoCA: a brief screening tool for mild cognitive impairment. *J Am Geriatr Soc*. 2005;53:695–9. doi:10.1111/j.1532-5415.2005.53221.x.
5. Yesavage JA, Sheikh JI. Geriatric Depression Scale (GDS) Recent evidence and development of a shorter version. *Clinical Gerontologist*. 1986;5(1-2):165-173. doi:10.1300/j018v05n01_09.
6. Kanis JA, Oden A, Johansson H, Borgström F, Ström O, Mccloskey E. FRAX® and its applications to clinical practice. *Bone*. 2009;44(5):734-743. doi:10.1016/j.bone.2009.01.373.
7. Podsiadlo D, Richardson S. The Timed “Up & Go”: A Test of Basic Functional Mobility for Frail Elderly Persons. *Journal of the American Geriatrics Society.* 1991;39(2):142-148. doi:10.1111/j.1532-5415.1991.tb01616.x.
8. American Geriatrics Society 2015 Updated Beers Criteria for Potentially Inappropriate Medication Use in Older Adults. *Journal of the American Geriatrics Society*. 2015;63(11):2227-2246. doi:10.1111/jgs.13702.
