# Supplementary material for: Geriatric Assessment in a Primary Care Environment: A Standardized Patient Case Activity for Interprofessional Students
Source: MedEdPORTAL. 2019 Oct 18;15:10844. doi: 10.15766/mep_2374-8265.10844 (PMC6944254; doi:10.15766/mep_2374-8265.10844)
Supplement: Supplementary file 1 — A. Logistics.docx B. Case Briefing.docx C. Student Instructions.docx D. IPE Feedback Rubric.docx E. SP Recruiting Criteria.docx F. SP Case Development Tool.docx G. Faculty Instructions and Debriefing Guide.docx H. Potential Discipline-Specific Learning Objectives.docx [file mep-15-10844-s001.zip › B. Case Briefing.docx]

**Appendix B: Case Briefing**

PART 1: FEMALE PATIENT

New Patient Intake Information

Name: Jo Smith

Gender: Female

Height: 5 feet, 5 inches

Weight: 115 pounds

Reason for Visit:

New patient visit to establish care with a provider; previous physician in private practice retired. Needs medications refilled. Had a recent fall. Patient has questions about a previous abnormal lab value that was marked as “high” from a year ago. Patient also has questions about a recent DEXA scan that was done at a community health event, with T-scores: L spine -2.2, left hip -2.4, and right hip -2.0. Patient completed a Geriatric Depression Scale screening in the waiting room.

Chronic Health Conditions:

● Hypertension

● Atrial Fibrillation

● Heart Attack

● Weak Bones

● Kidney Disease

● TIA

● Macular Degeneration

Hospitalizations and Surgeries:

● Heart attack

● TIA

● Appendectomy as a child

● Hysterectomy for abnormal bleeding at the age of 34

Allergies: Penicillin, erythromycin

Medications:

● Baby aspirin

● Warfarin

● Lisinopril

● Hydrochlorothiazide

● Potassium Chloride

● Calcium and Vitamin D

● Multivitamin

● Simvastatin 10 mg at bedtime

● Tylenol PM for sleep nightly

● Oxybutynin 5 mg twice a day

● Advil

● BenGay

● Red yeast rice supplement

● Metoprolol

— The patient brought two pills to show you. The patient stopped taking them after picking up the last refill because they looked different than in the past.

● Had historically been oblong, white, scored with the number 166.

● The most recent pills were pink, round, scored, with the imprint letter M and the number 32

---------------------------------------------------------------------------------------------------------------------

PART 2: MALE PATIENT

New Patient Intake Information

Name: Joe Smith

Gender: Male

Height: 5 feet, 8 inches

Weight: 135 pounds

Reason for Visit:

New patient visit to establish care with a provider; previous physician in private practice retired. Needs medications refilled. Had a recent fall. Patient has questions about a previous abnormal lab value that was marked as “high” from a year ago. Patient also has questions about a recent DEXA scan that was done at a community health event, with T-score: L Spine -2.2, left hip -2.4, and right hip -2.0. Patient completed a Geriatric Depression Scale screening in the waiting room.

Chronic Health Conditions:

● Hypertension

● Atrial Fibrillation

● Heart Attack

● Weak Bones

● Kidney Disease

● TIA

● Macular Degeneration

Hospitalizations and Surgeries:

● Heart attack

● TIA

● Appendectomy as a child

Allergies: Penicillin, erythromycin

Medications:

● Baby aspirin

● Warfarin

● Lisinopril

● Hydrochlorothiazide

● Potassium Chloride

● Calcium and Vitamin D

● Multivitamin

● Simvastatin 10 mg at bedtime

● Tylenol PM for sleep nightly

● Oxybutynin 5 mg twice a day

● Advil

● BenGay

● Red yeast rice supplement

● Metoprolol

— The patient brought two pills to show you. The patient stopped taking them after picking up the last refill because they looked different than in the past.

● Had historically been oblong, white, scored with the number 166.

● The most recent pills were pink, round, scored, with the imprint letter M and the number 32
