# Supplementary material for: Geriatric Assessment in a Primary Care Environment: A Standardized Patient Case Activity for Interprofessional Students
Source: MedEdPORTAL. 2019 Oct 18;15:10844. doi: 10.15766/mep_2374-8265.10844 (PMC6944254; doi:10.15766/mep_2374-8265.10844)
Supplement: Supplementary file 1 — A. Logistics.docx B. Case Briefing.docx C. Student Instructions.docx D. IPE Feedback Rubric.docx E. SP Recruiting Criteria.docx F. SP Case Development Tool.docx G. Faculty Instructions and Debriefing Guide.docx H. Potential Discipline-Specific Learning Objectives.docx [file mep-15-10844-s001.zip › C. Student Instructions.docx]

**Appendix C: Student Instructions**

**STUDENT INSTRUCTIONS:**

As found on the learning management site, there are discipline-specific learning objectives. In addition, there are also interprofessional learning objectives listed below.

**Interprofessional Learning Objectives:**

By the end of this activity, learners will be able to:

1. Work collaboratively with others who provide care to deliver preventive and/or health services.
2. Describe at least one aspect of another profession’s roles/responsibilities or scope of practice that you did not know prior to the activity.
3. Communicate discipline-specific knowledge to other members of the healthcare team with confidence and clarity.

For this session, you will be interacting with students from other disciplines. You will need to budget your time to be sure that all members of your team have a chance to contribute. Respect the contributions that each discipline can make and encourage quiet team members to share their insights.

If there is physical examination component that you wish to evaluate, ask the patient for permission.

Activity:

1. Based on case information you will be initially provided, decide what additional information you would like to obtain from the patient.
2. Decide as a team **WHO** is best suited to do **WHAT**
3. Engage in discipline-specific assessments of the patient
4. As a team of healthcare professionals, decide what some next steps should be for the patient’s care
5. Deliver your care plan to patient and provide appropriate patient education
6. Debrief with facilitator

Approximate Time Allotment:

● 1:30-2:00 Introductions; pre-brief to discuss what additional information each discipline will seek. **WHO** will do **WHAT**?

● 2:00-4:00 Discipline-specific assessments/care plan/patient education. Divide time equally across disciplines.

● 4:00-4:30 Debrief with facilitators/co-facilitators
