# Supplementary material for: Geriatric Assessment in a Primary Care Environment: A Standardized Patient Case Activity for Interprofessional Students
Source: MedEdPORTAL. 2019 Oct 18;15:10844. doi: 10.15766/mep_2374-8265.10844 (PMC6944254; doi:10.15766/mep_2374-8265.10844)
Supplement: Supplementary file 1 — A. Logistics.docx B. Case Briefing.docx C. Student Instructions.docx D. IPE Feedback Rubric.docx E. SP Recruiting Criteria.docx F. SP Case Development Tool.docx G. Faculty Instructions and Debriefing Guide.docx H. Potential Discipline-Specific Learning Objectives.docx [file mep-15-10844-s001.zip › D. IPE Feedback Rubric.docx]

**Appendix D: IPE Feedback Rubric**

| Parameter Assessed | 1 | 2 | 3 |
| --- | --- | --- | --- |
| Collaborative  Teamwork | Dismissive of other team members’ roles, knowledge, and/or contributions to healthcare | Respectfully receives input, ideas, and/or opinions from other disciplines | Actively seeks to involve other disciplines by encouraging others to get involved or asking questions to learn from them |
| Roles | Unable/unwilling to articulate anything learned from another discipline and/or ways in which this session differed by having interprofessional contributions rather than being solely uniprofessional | Limited ability to articular contributions of other disciplines. Limited or constricted viewpoint of other disciplines | Articulates something learned from another discipline and/or ways this session was different by having interprofessional contributions rather than being solely uniprofessional |
| Communicate with Confidence | Is hesitant to contribute to discussion/activity and may need prompting | Contributes to discussion/activity with appropriate level of confidence and humility | Overly confident to the point that student appears arrogant and/or tends to dominate the activity |
| Communicate with clarity | Does not engage in the activity to share discipline-specific information unless prompted | Shares information but uses discipline-specific terminology/jargon such that others may feel excluded | Shares information AND avoids using jargon and/or takes care to explain meanings to others. |
| Effective Communication | Is hesitant to contribute to discussion/activity and/or dominates communication to the detriment of others | Contributes to discussion/activity with appropriate verbal and non-verbal skill. May need occasional prompt. May use occasional jargon. | Effectively uses verbal and non-verbal skills throughout activity. Does not use jargon and uses patient-friendly language |
