# Supplementary material for: Geriatric Assessment in a Primary Care Environment: A Standardized Patient Case Activity for Interprofessional Students
Source: MedEdPORTAL. 2019 Oct 18;15:10844. doi: 10.15766/mep_2374-8265.10844 (PMC6944254; doi:10.15766/mep_2374-8265.10844)
Supplement: Supplementary file 1 — A. Logistics.docx B. Case Briefing.docx C. Student Instructions.docx D. IPE Feedback Rubric.docx E. SP Recruiting Criteria.docx F. SP Case Development Tool.docx G. Faculty Instructions and Debriefing Guide.docx H. Potential Discipline-Specific Learning Objectives.docx [file mep-15-10844-s001.zip › E. SP Recruiting Criteria.docx]

**Appendix E: SP Recruiting Criteria**

1. Recruitment Criteria
   1. Standardized patients were identified and selected among a pre-existing pool of standardized patients employed at our institution. Most SPs were familiar with the premise of the case and had been actors in previous years when the educational activity was conducted for medical students only.
2. SP Training Methods:
   1. Methods used for training:
      1. Sixteen individuals were trained as standardized patients (SP) for this case. The SPs were provided with an instructional document (**Appendix F**: SP Training and Instruction) two weeks in advance of on-site training which was conducted by an interprofessional group of faculty. During the training session, faculty across the represented professions read the instructional document aloud with the SPs (Appendix F: SP Training and Instruction). Faculty provided a thorough interpretation of the case, and answered any questions that were raised. In addition, faculty demonstrated how the SPs should behave (e.g. performance with mobility, etc.) and respond when student learners performed specific examinations or asked certain questions pertinent to the case. The SPs practiced these same behaviors and movements with feedback from the faculty.
   2. Data to support the content of the materials:
      1. Faculty across all represented professions contributed to development of the case and training of the SPs to ensure accuracy.
   3. Data to support the reliability of any related checklists or rating scales
      1. The rating scale was applied to students by facilitators rather than SPs. It was created for student feedback based upon similar scales that have previously been used to assess team interactions. Specifically, the TOSCE (team objective structured clinical examination), created by McMaster University available at<https://fhs.mcmaster.ca/tosce/en/administration_checklist.html> was used as a starting point, along with a modified TOSCE tool developed by Lie et al., 2015.^1^ We further modified the instrument by decreasing the number of items being assessed as well as providing descriptors for each of three possible ratings that could be given and taking care to align the instrument with the interprofessional learning objectives.
   4. Methods used to assess portrayal, feedback, and/or checklist accuracy
      1. Facilitators attended a faculty development training session that focused on explaining the competencies that were being assessed, applying the feedback rubric to various situations, and debriefing.

References:

1. Lie D, May W, Richter-Lagha R, Forect C, Banzali Y, Lohenry K. Adapting the McMaster-Ottawa scale and developing behavioral anchors for assessing performance in an interprofessional team observed structured clinical encounter. Med Educ Online. 2015;20:26691.
