# Supplementary material for: Geriatric Assessment in a Primary Care Environment: A Standardized Patient Case Activity for Interprofessional Students
Source: MedEdPORTAL. 2019 Oct 18;15:10844. doi: 10.15766/mep_2374-8265.10844 (PMC6944254; doi:10.15766/mep_2374-8265.10844)
Supplement: Supplementary file 1 — A. Logistics.docx B. Case Briefing.docx C. Student Instructions.docx D. IPE Feedback Rubric.docx E. SP Recruiting Criteria.docx F. SP Case Development Tool.docx G. Faculty Instructions and Debriefing Guide.docx H. Potential Discipline-Specific Learning Objectives.docx [file mep-15-10844-s001.zip › F. SP Case Development Tool.docx]

**Appendix F: SP Case Development Tool**

Date: November 5, 2018

Primary Case Author: Peter Lewis, Sam Faber, Lisa Sherwood

Secondary Case Author: Kelly Karpa, RPh, PhD, Megan Brightbill RDH, MEd, PHDHP, Gina Fox, OTD, OTR/L, Shawnee Kelly, MS, RDN, LDN, FAND, Matthew Walko, MS, DPT

Standardized Patient Educator: Kelly Karpa, Angela Salvadia, Matt Walko

Name of Case: The Interprofessional Comprehensive Geriatric Patient Assessment

Name of educational and or assessment activity: Geriatric Assessment in a Primary Care Environment: A Standardized Patient Case Activity For Interprofessional Students

Patient Name: Jo or Joe Smith

Chief Complaint: establish care with a provider

Most likely Diagnosis and Differential with rationale from history and/or physical exam:

Osteoporosis: FRAX assessment

Malnutrition: patient history, weight loss

Polypharmacy: medication reconciliation and optimization

Cognitive issues: cognitive screening

Poorly fit dentures: patient history

Challenge question: Develop a care plan for a geriatric patient through effective interprofessional collaboration.

Domains:

⛝ Professionalism

⛝ Communication and Interpersonal skills

⛝ Medical History

⛝ Physical exam

⛝ Shared Decision Making

⛝ Patient Education

⛝ Clinical Reasoning

☐ Documentation

☐ Handoff

☐ Presentation

☐ Other:

Type and level of learners: OT students = 4^th^ year; PT students = 5^th^ year; RN students = 3^rd^ year; MD students = 2^nd^ year; RD = internship year; Dental = 2^nd^ year; Pharm = 6^th^ year

Case Objectives:

By the end of this activity, learners will be able to:

1. Work collaboratively with others who provide care to deliver preventive and/or health services.
2. Describe at least one aspect of another profession’s roles/responsibilities or scope of practice that you did not know prior to the activity.
3. Communicate discipline-specific knowledge to other members of the healthcare team with confidence and clarity.

| SETTING: | Outpatient, primary care office |
| --- | --- |
| PATIENT PROFILE: | |
| Age range | 65-90 years old (our standardized patient was said to be 82 years old) |
| Religious/spiritual background | Used to be very active in the church |
| Sex (e.g., male, female, intersex, transwoman, transman) | Male or Female |
| Sexual Orientation (e.g., heterosexual, lesbian, gay, bisexual, pansexual, queer, asexual) | Heterosexual |
| Gender expression (e.g., man, woman, gender queer) | Not specified |
| Race/ethnicity: | All may be used |
| Physical description (e.g., BMI, height range) | Female: 5 ft 5 in, 115 lbs  Male: 5 ft 8 in, 135 lbs |
| Physical limitations | Patient has been feeling stiff since a recent fall |
| Patient appearance (e.g., disheveled, hospital gown, business casual, casual) | Casual |
| Moulage + location (e.g., none, bruises, scars, body piercing, tattoos) | None |
| Affect (e.g., pleasant, cooperative) | Slightly confused at times; admits memory is not as good as it used to be |
| Family group (e.g., who is family, who they live with) | No children, spouse died 6 months ago, relies on niece for transportation |
| Education | High school education |
| Level of health literacy | Minimal; patient should prompt students to use layman’s terms if they start using medical jargon or abbreviations |
| Employment, if any - present and past, noting any current stresses | Worked in the township tax office for many years before retiring at the age of 62. |
| Home/homeless - type of dwelling, number of stories, owned or rented | Lives in ranch-style house that is paid for; there are steps to get to the washing machine in the basement. |
| Financial situation- any current stresses | The electric company just sent a notice about being behind in payment, but the patient doesn’t believe that because the bills get paid regularly |
| Insurance Status (e.g., un/under/insured, public/private, HMO/PPO) | Not specified |
| Habits (i.e., diet, exercise, caffeine, smoking, alcohol, drugs) | Haven’t smoked in decades |
| Activities (i.e., hobbies, sports, clubs, friends) | Recently none. Stopped most activities after death of spouse 6 months ago. Had enjoyed oil painting when vision was better |
| Typical day - what is the usual daily routine | Niece provides transportation to appointments when she know about them, but the patient manages own healthcare and make appointments. Niece also takes patient to the grocery store when asked, but patient really hates being dependent on her. Patient eats 2-3 times per day: usually hot tea and a piece of fruit for breakfast. Lunch is usually toast with butter or cheese slice or ½ sandwich with hot tea. If hungry in the afternoon, patient eats whatever is around the house – usually saltine crackers or butter cookies. One of neighbors brings a casserole dish about once a week that is divided up into many small meals that is eaten for supper. Patient used to walk around the neighborhood regularly, but has not been feeling steady the past couple of months. Since no longer driving, the patient has not been going to church due to being dependent upon others for rides. Patient and spouse had been very active in your church; the minister and some members stop at the house occasionally to visit. |

| CASE INFORMATION | |
| --- | --- |
| Chief Concern: What the patient will say when greeted by the student. The patient’s primary reason for seeking medical care often stated in his/own words. | “I am here to get a new doctor, my previous primary care physician retired, and I need some of my medications refilled. I also fell recently.” |
| Additional Concerns: Other, if any, concerns the patient has today (i.e., symptoms, requests, expectations, etc.) that will become part of set agenda. | “I was told I had a “high” lab value last year but I’m not sure what test it was and if I was supposed to do anything for it. It said SCr, 1.9.”    “I had a bone scan done at the community health fair, I’m pretty sure I have weak bones because someone told me that a while ago.”    “Here is the survey that I completed in the waiting room.”    “My niece doesn’t think I should be driving anymore and she is worried about leaving me alone.”    “I have lost some weight recently and my clothes seem a lot loose.r” |
| THE PATIENT STORY: The SP will be asked to tell their symptom story and the personal and emotion impact for each of their concerns. You will want to write this is the patient voice. The symptom story should be able to answer this question: “Tell me more about [chief concern/additional concern], starting at the beginning and bringing me up to now.”    The personal context should be able to answer questions concerning the broader personal/psychosocial context of symptoms, especially the patient beliefs/attributions.    The emotional context should be able to ask how are you doing with this, how does this make you feel, how has this affected you emotionally? IMPACT: How has this affected your life? How has this been for your family? | “My spouse passed away 6 months ago. S/he used to do all the cooking and shopping. Now I live alone. Today I want to meet my new doctor and get some blood work done as I am overdue for my INR check by a couple of months. I also stopped taking my metoprolol because the pills looked differently when I picked them up at the pharmacy the last time, and I ran out of simvastatin a couple of weeks ago.  I am an 82 year old male/female who is new to the practice as my previous physician has retired. I have lost weight over the past few months. (If female, I did weigh 128 pounds 6 months ago; now I am down to 115 pounds and I am 5 feet 5 inches in height. If male, I weighed 145 pounds 6 months ago; now, I weigh 135 pounds and I am 5 feet 8 inches tall.)  It has been 6-9 months since my last doctor’s visit and I was used to being seen every 4-6 months. It’s been a year since I have had general blood work. I usually get my blood thinner bloodwork checked monthly, but that’s been a while, too.  I recently went to a walk-in clinic for a urinary tract infection and when they asked me what medications I was on. I forgot that I was on a blood thinner. I am getting more forgetful which makes me worried because my mother died at 81 and she was senile the last few years of her life.    My niece (late brother’s daughter) doesn’t think I am taking care of myself, and she is worried about me being alone. I’m upset because she doesn’t think I should be driving but I guess she is right. She drove me here today and is out in the waiting room.” |
| HISTORY OF PRESENT ILLNESS: | |
|  | |
| Onset (when; gradual or sudden) | Gradual |
| Setting (what was going on or where was patient when symptoms first noticed?) | NA |
| Duration (how long) | NA |
| Time relationships (frequency, constant or intermittent) | NA |
| Location | NA |
| Radiation | NA |
| Quality | NA |
| Amount | NA |
| Aggravated by what | NA |
| Relieved by what | NA |
| Associated with what | NA |
| Attitude (what does the patient think is the problem, and how does he/she feel about it) | A little confused and worried about memory |
| Overall course | NA |
| REVIEW OF SYSTEMS: Significant positives and negatives | |
| General: your clothes are fitting much looser so you think that you’ve lost weight. No fever, chills or sweats. Not very hungry. **Be sure to mention: that you look so pale. You have dark circles under your eyes, deep hollows over your temples - you barely recognize yourself in the mirror these days** (these are clues suggestive of malnutrition)**.**  **Eyes:** macular degeneration with some visual loss in the right eye. You see the eye doctor every 6 months.  **ENT:** Hearing is not as good as in the past, but turning up the TV helps. **Be sure to mention: Your mouth is dry and you often take dentures out when eating because they slides around too much** (this is a clue that your dentures do not fit properly)**.**  **Cardiovascular:** Your heart seems to beat faster and you’ve been a little winded with some of your normal activities like doing the laundry. Your neighbor has been helping with the laundry.  **GU:** good bladder control except during a previous infection but now you are much better.  **MSK**: Hips and knees bother you. They are especially stiff when you get up in the morning and after sitting for a while. **Be sure to mention that your bones are sticking out on your chest, shoulders, and arms since you lost weight. Be sure to also mention that you look so pale in the mirror** (these things are suggestive of malnutrition)**.** After this recent fall you’ve been using Bengay 2-3 x per day on your left hip and buttock where you landed.  **Dermatologic**: Lots of bruising lately.  **Psychiatric:** Completed the Geriatric Depression Scale: Short Form in waiting room. You do have some trouble sleeping and have been taking Tylenol PM regularly at bedtime.  **Neurologic**: Your strength and energy seem to be decreasing, and you don’t get around as easily as you did when you were younger. Your legs ache and tingle and burn, and it’s especially bad when you lay down at night. You sleep a lot and are thankful that your neighbor has been helping with the laundry because you can’t make it up and down stairs very well. You can’t open jars in the kitchen | |
|  | |
| Past medical history - **If asked for more details about chronic health conditions, you may plead “I don’t remember”** | |
| Medication allergies (Name and reaction) | · Penicillin (hives)  · Erythromycin (upset stomach) |
| Environmental allergies (Name and reaction) | None |
| Illnesses | · Hypertension  · Atrial fibrillation  · Heart attack  · Weak bones  · Kidney disease  · TIA  · Macular degeneration |
| Vaccinations | · Tdap  · Pneumovax 23 (at age 60)  · Prevnar 13 |
| Surgeries | · Appendectomy as a child  · Hysterectomy for abnormal bleeding at the age of 34 (if female) |
| Accidents/ injuries/ trauma | · Recent fall resulting in minor bruising  · No major/traumatic injuries or trauma |
| Hospitalization | · Heart attack  · TIA |
|  | |
| Inclusive sexual and reproductive history | |
| Sexual practices  Sexual partners  Protection: Use of safer sex practices  Use of birth control if appropriate  Risk of intimate partner violence | Not applicable |
| Ob/GYN HISTORY | Age of onset of menses: NA  Age of menopause: NA  Number of pregnancies: 0  Number of live births: 0  Number of miscarriages: 0  Number of abortions 0 |
| Medications | ● Baby aspirin  ● Warfarin  ● Lisinopril  ● Hydrochlorothiazide  ● Potassium chloride  ● Calcium and Vitamin D  ● Multivitamin  ● Simvastatin 10 mg at bedtime for cholesterol  ● Tylenol PM for sleep nightly  ● Oxybutynin 5 mg twice daily  ● Advil for hip and knee stiffness. Uses 4-6 pills a day as needed on an empty stomach  ● BenGay 2-3x per day since the fall  ● Red yeast rice supplement twice daily  ● Metoprolol tartrate |
| Immunizations | Tdap  Pneumovax 23  Prevnar 13 |
| Tobacco products:  X Cigarettes  □ Cigar  □ Pipe  □ Chew  □ E-cigarettes | Previous smoker – only ever smoked a few cigarettes, but it has been many years in the distant past  □ Never  ✓ Past –years in the distant past  □ Current  o Occasional consumption # of years: All may be used |
| Alcohol  □ Beer  □ Wine  □ Liquor  □ Other | ✓ Never  □ Past- year started/year quit  □ Current  o Quantity # of years |
| Drugs  □ Weed  □ Cocaine  □ Heroin  □ Meth  □ Other  □ IV  □ Inhalants  □ Other | ✓ Never  □ Past- year started/year quit  □ Current  o Quantity # of years |
| Diet (describe) | The patient reports eating 2-3 times per day. Breakfast consists of fruit and hot tea. Lunch is usually toast with butter or cheese slice or half of a sandwich with hot tea. Sometimes the patient eats saltines or butter cookies if he/she gets hungry during the afternoon. A neighbor brings a casserole dish about once a week that is divided up into many small meals for supper. |
| Exercise (describe) | The patient used to walk around the neighborhood regularly, but has been feeling less steady the past couple of months. |
| List any other important social history or information important to this case | The patient does not have a computer nor do they know how to use one.  The patient is no longer driving at the request of his/her niece despite not having any accidents.  The patient is frustrated with becoming more dependent on others. |
| Family history |  |
| Mother, Father, Siblings, Grandparents, and other significant findings. | Father died of a heart attack in his 60’s  Mother died at 81 with senile dementia and osteoporosis that she had for several years. She fell and broke her hip at age 78 and declined significantly after that fall.  Brother died at 72 of a heart attack. He had a stroke the year before that. His daughter lives nearby and provides transportation. The patient does not have any children. |
| Physical Exam    The students may ask the SP to stand up, walk, turn around and sit down to check balance and ambulation. SPs should perform this task exceedingly slowly: slow to stand, walk slowly with feet/legs wide apart to maintain balance, and sit slowly.    Students may also ask the SP to participate in a cognitive screening test. SPs should perform incorrectly on delayed memory recall (e.g. only recalling two out of three words) and incorrectly on the visuo-spatial sections (e.g. unable to correctly draw “10 past 11” on a clock; incorrectly points hands at the numbers 10 and 11).    The students will be given the results of the Geriatric Depression Scale: Short Form screening tool. There will be 6 answers that are suggestive of depression:  Have you dropped many of your activities and interests? (Yes). You can mention that it has become difficult to see, hear, and you feel unsteady  Do you often get bored (Yes). You can mention no longer going to church, you see friends less, and you have stopped your hobby of oil painting.  Do you prefer to say at home rather than going out and doing new things (Yes). See the reasons above.  Do you feel you have more problems with memory than most? (Yes). And, your mother had dementia and that scares you.  Do you feel full of energy? (No).  Do you think most people are better off than you are (YES). They all seem to be younger, with less difficulty getting around, still have their spouse, etc.      The SP should be certain to mention these things in layman’s terms because these are clues to the dietitian and dental students:  -Facial features: dark circles under eyes  -Mouth: loose fitting dentures; dry oral mucosa  -Musculoskeletal: bones on shoulders and chest are visibly sticking out  -Skin: pale | |
| PHYSICAL EXAM FINDINGS |  |
| 1) Written in layman’s terms | The patient is sitting comfortably. |
| 2) General appearance- affect, appearance, position of patient at opening (i.e. sitting, laying down, holding abdomen etc.) | At times, the patient seems to have memory difficulties. |
| 3) Vital signs | Will be whatever is normal for the SP (e.g. a student will take blood pressure, pulse, and temperature) |
| 4) Specific findings and affect | a. Psychiatric- according to geriatric depression scale the patient has mild depression (6 answers suggestive of depression); the patient is confused about his/her medications |
| 5) Response to certain physical movements | The students may ask the SP to stand up, walk, turn around and sit down to check balance and ambulation. SPs should perform this task exceedingly slowly: slow to stand, walk slowly with feet/legs wide apart to maintain balance, and sit slowly. |
| DIAGNOSIS AND DIFFERENTIAL |  |
| Diagnosis with support from positive and negative history and PE findings | Osteoporosis; patient reported having a DEXA score done, FRAX is positive with a risk of hip fracture >3% for both female and male patients    Malnutrition: the patient lost almost 10% of his/her body weight and bones are protruding under the skin due to lack of nutritional intake from a “tea and toast diet”    Polypharmacy: drug interactions between simvastatin and red yeast rice; Tylenol PM and oxybutynin are contributing to the dry mouth; potassium chloride and oxybutynin were for acute issues and may not be necessary anymore.    Poorly fitting dentures: based on dental images and patient comments    Cognitive issues/mild depression: unable to complete all items on cognitive screen correctly; geriatric depression scale rating of 6 |
| Differential with support from positive and negative history and PE findings | Osteoporosis: rule out osteopenia utilizing the FRAX tool    Malnutrition: other causes of unexplained weight loss could be investigated    Polypharmacy: n/a    Poorly fitting dentures: must rule out patient nonadherence or misuse    Cognitive issues/mild depression: scores less than 5 indicate no depression, scores greater than 8 indicate more serious depression; may be situational due to the loss of spouse and ability to drive |
| MANAGEMENT OR DIAGNOSITIC PLAN | Osteoporosis: The patient is a candidate for treatment based on FRAX risk assessment. The patient is already taking calcium and vitamin D; therefore, a bisphosphonate may be warranted.    Malnutrition: recommend referring patient for resources such as food stamps or meals on wheels to improve overall diet; could also provide nutritional education or refer to dietician    Polypharmacy: deprescribe unnecessary medications such as potassium chloride and oxybutynin. Counsel the patient that oxybutynin and Tylenol PM are contributing to dry mouth and possibly cognitive issues.    Poorly fitting dentures: refer to dentistry for a full work up    Cognitive issues/mild depression: encourage patient to seek counseling services; consider appropriateness of antidepressant; refer to psychiatry if necessary |
| PROFESSIONALISM ISSUES OR CHALLENGES: | During the simulation, it can be challenging for students to budget their time appropriately such that each discipline is able to do an assessment of the patient from their discipline-specific perspective to ensure equal contributions from all disciplines to the patient’s care. |
