# Supplementary material for: Geriatric Assessment in a Primary Care Environment: A Standardized Patient Case Activity for Interprofessional Students
Source: MedEdPORTAL. 2019 Oct 18;15:10844. doi: 10.15766/mep_2374-8265.10844 (PMC6944254; doi:10.15766/mep_2374-8265.10844)
Supplement: Supplementary file 1 — A. Logistics.docx B. Case Briefing.docx C. Student Instructions.docx D. IPE Feedback Rubric.docx E. SP Recruiting Criteria.docx F. SP Case Development Tool.docx G. Faculty Instructions and Debriefing Guide.docx H. Potential Discipline-Specific Learning Objectives.docx [file mep-15-10844-s001.zip › G. Faculty Instructions and Debriefing Guide.docx]

**Appendix G: Faculty Instructions and Debriefing Guide**

**For the Faculty**

At today’s geriatric assessment, there will be students from not only the MD program but also from nursing, occupational therapy, physical therapy, dental hygiene, pharmacy, and dietitian programs.

At the beginning of the session, please ask the students to introduce themselves and the discipline they represent. To break-the-ice, ask them to also share, “If I were the title of a song, I would be….” or “If I could have dinner with anyone, dead or alive, it would be…”, or another topic of your choosing.

Approximate Time Allotment:

● 30 minutes: Introductions. Hand out case. Students should pre-brief to discuss what additional information each discipline will seek and decide **WHO** will do **WHAT** when the patient enters the room.

● Two hours: Discipline-specific assessments. Develop a collaborative care plan as a healthcare team. Deliver the care plan to patient and provide patient education as needed. Divide time equally across disciplines. You will guide this segment, perhaps serve as a time-keeper, but you will not lead it.

● 30 minutes: Debrief with facilitators/co-facilitators

Following introductions to team mates, students will review the background information (Chart Notes) about the patient they will be encountering. Ask the students to discuss what they would like to do/explore with the patient. **Who** on the team will do **what**? **How** will they divide up tasks so that everyone contributes? They should have a plan, and someone will need to monitor the time, so that one student/discipline doesn’t dominate the activity and everyone has a chance to contribute to the activity.

Debriefing

The structure of the debrief uses the Gather (How do you think that went?), Analyze (talk about what happened/what was observed), Summarize (The things that you have told me you learned today are…) approach. Facilitators are encouraged to frame their questions using the Advocacy-Inquiry approach.

Talking points relevant to the case that Facilitators should use to debrief with student

**Medication Issues**:

● The importance of the brown bag of medications at the initial visit at least to get an accurate list of medications/doses. How can students find this information if the patient and the electronic health record doesn’t have an accurate list? (e.g. call the pharmacy)

● The importance of up to date medication lists (drugs/dietary supplements and dosages) in patient’s wallets (and in the electronic health record).

● The benefits of weekly prefilled pill box organizers (e.g. Medisets). Some pharmacies will prepare these for patients.

● The need to simplify the medication list whenever possible.

o Once daily medications when possible (metoprolol succinate instead of tartrate or use a statin that works as well in the morning)

● Consider removing medications started for past problems that should have resolved (potassium chloride, oxybutynin) - get in the habit of “deprescribing”

● Be aware of the Beers list of drugs in the frail elderly—like medications with anticholinergic properties (e.g. diphenhydramine or oxybutynin) where memory issues and confusion are a concern. Discuss the Beers pocket guide and other resources for medication safety in older adults. Emphasize this is ONLY a guide and does not replace experience of the practitioner.

● Beware of drug-drug risks/interactions like aspirin and ibuprofen (chronic ibuprofen may negate antiplatelet benefits of aspirin; warfarin and ibuprofen (increased risk of bleeding); or warfarin and BENGAY (methyl salicylate) (potential for increased anticoagulant action); or potassium and lisinopril (increased risk of hyperkalemia); or red yeast rice and statins (increased risk of rhabdomyolysis).

● Beware of drug-disease risks to kidneys with combinations of nonsteroidal anti-inflammatory drugs (NSAIDs)/diuretics/ angiotensin converting enzyme inhibitors (ACEIs); chronic kidney disease and potassium; or peptic ulcer disease history and NSAIDS (especially when taken on empty stomach)

● Based upon the info provided, students can calculate creatinine clearance (CrCl). Students should know that lisinopril is renally excreted. Based upon patient’s renal function and CrCl, students should discuss renal-dosing of medications.

● Consider the risks of forgetful patients taking anticoagulants. What are advantages and disadvantages of warfarin (e.g. advantage: longer acting than direct oral anticoagulants which may be beneficial in forgetful patients, inexpensive; disadvantage: periodic monitoring needed).

● How to identify pills that patients may bring to office visits since many different manufacturers may make the same medication, but the pills can look differently (different colors, shapes, imprints). Googling “pill identifier” will take students to<https://www.drugs.com/pill_identification.html> Students can reassure the patient that the correct medication (metoprolol tartrate 50 mg) was dispensed.

**Nutrition issues**

● The patient has lost close to 10% of body weight over the past six months

● Nutritionally, the patient is eating a “tea and toast” diet, which places him/her at risk of B12 deficiency. A B12 deficiency could also affect strength, sense of well-being, lead to peripheral neuropathies (fall risk)/burning/tingling, and contribute to memory problems.

● The patient may not be hungry/eating much on account of: cost, transportation difficulties, dry mouth (due to anticholinergic effects of medications), pain associated with poorly-fitting dentures, and/or depression. Encourage students to think of other team members/services that might be helpful if cost or transportation difficulties for getting to supermarket are an issue (e.g. social work might be able to help get the patient set up for food stamps if the patient meets criteria; Meals On Wheels could deliver a hot meal daily, Monday through Friday).

● The patient describes signs/symptoms consistent with moderate malnutrition:

o Facial features: temples, slightly hollowing; orbital area, dark circles; buccal area, face/cheeks appear flat

o Mouth: oral cavity, loose fitting dentures; dry oral mucosa

o Musculoskeletal: clavicle/pectoralis area – visibly prominent; shoulder/deltoid, acromion process is visible upon inspection and palpation

o Skin: mild pallor noted

**Oral Health issues**

● The patient is taking several medications that may be contributing to dry mouth (e.g. diphenhydramine, oxybutynin) which may lead the patient to select food options that are easier to swallow; perhaps these medications can be discontinued.

● The patient’s bone density or weight loss could contribute to ill-fitting dentures. Changes in the ridge of bone that the denture was initially made to fit can be related to bone loss and/or weight loss. New dentures may be needed.

**Depression**

● Based upon the results of the Geriatric Depression Scale that the patient completed, s/he may have mild depression (5-8 items indicates mild depression). This could be situational following death of spouse, loss of driving privileges, etc. but should be explored further with the patient.

● Given changes in the patient’s social/personal engagement (death of spouse, loss of driving, etc.), it is reasonable to explore the relationship between social engagement and health and well being.

**Osteoporosis Risk**

● The FRAX tool is available at: <https://www.sheffield.ac.uk/FRAX/tool.aspx?country=9>

o Provide guidance for using the FRAX tool to determine if patient meets criteria for initiating treatment for low bone mass.

o FRAX is an online validated risk calculator assessing osteoporosis fracture risk- need age, gender, weight, height, family and personal history of fractures, alcohol and smoking history, steroid use, and most recent DEXA scan lowest T-Score information placed into a calculator, and the results given are 10 year risk for any fracture vs 10 year hip fracture risk. If 10 year any fracture risk above 20% or hip fracture risk >3% there is an indication for starting treatment. Each group should access the online tool.

**Vital Signs and Other Foundational Assessments**

● Did someone verify patient’s name and date of birth?

● The patient’s vital signs should be checked (temperature, blood pressure, heart rate); blood pressure and heart rate may be elevated since patient has not been taking medications as prescribed

● Students may perform auscultation (heart, lung, bowel)

● Students should assess patient’s current interests, what is important to the patient, establish foundational information.

**Mobility**

● What factors indicate the patient is having some mobility issues (avoids stairs, has fallen)

o Students may assess range of motion, strength, sensation

o Based on the examination, students will consider appropriateness of assistive devices

● Perform the Timed Up and Go (TUG) Test

o A video link for this is available online<https://www.physio-pedia.com/Timed_Up_and_Go_Test_(TUG)>

● Assess transfer ability (sit-stand, bed-chair transfers)

● Role of physical therapy may overlap with occupational therapy

**Activities of Daily Living**

● The patient reports difficulty remembering to take medications and send bills. The patient reports being upset that his/her niece told him/her to stop using the stove since the patient says he/she only forgot to turn it off once.

● The patient reports feeling “unorganized” during the day and having trouble keeping track of everything.

● As far as other leisure activities, the patient has stopped attending church and no longer engages in oil painting. The patient has difficulty opening jars in the kitchen.

● A screening tool can be conducted to assess for cognitive function

**Vision**

● Given that the patient has macular degeneration and has had a recent fall, it is reasonable that students might choose to further explore this.

● A referral for a low vision evaluation by a specialist might be warranted.

**Patient Education (examples below)**

● Review medications with patient so s/he knows the reason for each, how/when to take it, and potential adverse effects.

● Review strategies to reduce fall risk in the home

● Review memory strategies, work simplification, organizational strategies for cognitive functioning in performing daily tasks.

● Review proper care of dentures

● Provide nutritional information to patient

**Interprofessional education discussion**

● It should be reassuring to students that they don’t need to “know it all”. There are other members of the team to rely on. Probe the following aspects pertaining to interprofessional competencies:

- Where do roles overlap? Is that good? Bad? Why?
- What was learned about another discipline that wasn’t previously known?
- How difficult was it to speak up and contribute discipline-specific knowledge today? Was there a hierarchy today? What have you experienced on healthcare teams with regard to hierarchy? In what ways does hierarchy affect patient care? What can students do to address hierarchies in healthcare when in clinics/wards/practice environments?
