# Supplementary material for: Geriatric Assessment in a Primary Care Environment: A Standardized Patient Case Activity for Interprofessional Students
Source: MedEdPORTAL. 2019 Oct 18;15:10844. doi: 10.15766/mep_2374-8265.10844 (PMC6944254; doi:10.15766/mep_2374-8265.10844)
Supplement: Supplementary file 1 — A. Logistics.docx B. Case Briefing.docx C. Student Instructions.docx D. IPE Feedback Rubric.docx E. SP Recruiting Criteria.docx F. SP Case Development Tool.docx G. Faculty Instructions and Debriefing Guide.docx H. Potential Discipline-Specific Learning Objectives.docx [file mep-15-10844-s001.zip › H. Potential Discipline-Specific Learning Objectives.docx]

**Appendix H. Potential Discipline-Specific Learning Objectives**

By the end of the session, learners will be able to:

**OT Learning Objectives**

1. Accurately implement, score and interpret the MOCA with the client
2. Identify at least 1 additional assessment tool they would complete to gain a more in depth understanding of the client’s cognitive deficits.
3. Communicate 2 recommendations/interventions based on assessment findings with the client and other team members.
4. Demonstrate effective communication with interdisciplinary team members and with the client.

**PT Learning Objectives:**

1. Correctly administer and interpret the timed-up-and-go test for balance and fall risk assessment
   1. Consider an alternative to this test, if appropriate
2. Identify up to 3 additional tests and measures that would contribute to a successful overall patient assessment, particularly regarding fall risk and fracture risk
3. Identify at least 2 interventions (including assistive device recommendations) that the patient can implement after this initial session, and coordinate with other members of the management team as appropriate
4. Successfully communicate findings and additional recommendations to the patient and other members of the management team

**MD Learning Objectives**

1. Perform a geriatric assessment including evaluating functional abilities, cognitive decline, age related physical changes, and appropriateness of medications in older adults.
2. Evaluate the impact of certain medications and polypharmacy on patient safety and fall risk in older adults.
3. Identify BEERS criteria and appropriate medication use in care of older adults.
4. Describe the timed up and go test and implement in a clinical encounter.
5. Employ screening tests for depression in a geriatric patient.
6. Employ screening tests for cognitive decline and implement in a patient encounter.
7. Employ the FRAX tool to assess fracture risk in a patient with osteopenia and calculate fracture risk

**RD Learning Objectives**

1. Demonstrate ability to conduct a patient history and diet interview.
2. Perform a nutrition focused physical examination.
3. Complete a nutrition assessment
   1. verify and interpret data
      1. anthropometric data
      2. biochemical data
      3. clinical data (medications, histories, review of systems, history & physical, etc.)
      4. dietary data
      5. nutrition focused physical exam findings
4. Determine high priority nutrition diagnosis
5. Determine appropriate nutrition care plan including intervention, monitoring, and evaluation.

**Dental Hygiene Learning Objectives**

1. Collect and analyze data to determine the general and oral health of the client
2. Develop and recommend an oral health treatment plan with realistic goals and evidence-based standards for care
3. Consult with medical professionals regarding the client’s overall health

**RN Learning Objectives**

1. Demonstrate ability to complete a focused physical assessment of an older patient

2. Identify safety issues related to polypharmacy in an older adult

3. Develop an interdisciplinary plan of care to address the holistic needs of an older adult patient
